# Effects of Activated Charcoal-Herb Extractum Complex on Antioxidant Status, Lipid Metabolites and Safety of Excess Supplementation in Weaned Piglets

**DOI:** 10.3390/ani9121151

**Published:** 2019-12-15

**Authors:** Liqi Wang, Lin Zhu, Limin Gong, Xin Zhang, Yubo Wang, Jianling Liao, Linfu Ke, Bing Dong

**Affiliations:** 1 State Key Laboratory of Animal Nutrition, China Agricultural University, Beijing 100193, China; wangliqi1224@cau.edu.cn (L.W.); zhulin1990s@163.com (L.Z.); gonglimin@cau.edu.cn (L.G.); zhangx0904@126.com (X.Z.); wangybcau@163.com (Y.W.); 2 Fujian Baicaoshaung Biotechnology Co., Ltd., Nanping 353200, China; fjbcsgs@163.com (J.L.); 13509529066@163.com (L.K.)

**Keywords:** activated charcoal-herb extractum complex, weaned piglets, antioxidant status, serum lipid metabolites

## Abstract

**Simple Summary:**

Weaning is the most significant event in the life of pigs and pigs must cope with the sudden disruption of social interaction with sows and litters and pressure to adapt to new environments that may impair the growth performance and health status of piglets. Weaning can induce oxidative stress and disturb the balance of lipid metabolism in pigs. In this study, we innovatively evaluated a complex of activated charcoal and Chinese herb extractum (CHC) in weaning pigs on antioxidant status, serum lipid metabolites and safety supplementation in weaning pigs. This complex combines functions of active charcoal in absorption of toxins and the Chinese medicinal herbs in antioxidant capacity. The results demonstrated a promising additive of this complex CHC in future applications. This complex may be a good nutritional supplement to use in swine production.

**Abstract:**

This study was aimed at evaluating the effects of activated charcoal-herb extractum complex (CHC) on antioxidant status, serum lipid metabolites and its safety supplement in weaning piglets. In experiment 1, a total of 216 piglets (Duroc × Landrace × Large White) weaned at 28 days of age with initial body weight of 8.55 ± 1.18 kg were assigned randomly to six treatment groups. each treatment group had six pens, with six pigs per pen. Pigs were fed a corn-soybean meal-based diet supplemented with 500, 1000, 1500 or 2000 mg kg^−1^ of CHC over two 14-d periods. Diets supplemented with 0 and 1000 mg kg^−1^ of montmorillonite (MMT) were set as the negative and positive controls, respectively. In experiment 2, pigs (*n* = 108) weaned at 28 days of age with initial body weight of 8.58 ± 0.04 kg were randomly assigned to three treatment groups. Each treatment group had six pens, with six pigs per pen. Pigs were fed a corn-soybean meal-based diet supplemented with 0, 1000 or 10,000 mg kg^−1^ of CHC over two 14-d periods. In experiment 1, on day 14, supplementation with CHC significantly decreased very low-density lipoprotein (VLDL) concentration while they decreased low-density lipoprotein (LDL) concentration on d 28, CHC at 500, 1000 or 1500 mg kg^−1^ significantly increase high-density lipoprotein (HDL) concentration. Supplementation with 500 or 1000 mg kg^−1^ CHC reduced serum malondialdehyde (MDA) concentration during the entire experimental period and increased the concentration of serum total superoxide dismutase (T-SOD) on d 14. CHC at 500 or 1000 mg kg^−1^ significantly reduced the liver MDA concentration and increased liver T-SOD concentration. In experiment 2, increased ADG was obvious during the first 14 days and the whole period in 1000 mg kg^−1^ supplemented pigs, similarly F: G was lowest in the first 14 days. There was no difference in growth performance, visceral index, haematological and serum biochemical parameters and visceral organs morphology between pigs fed 10,000 mg kg^−1^ of CHC and control. Together, 500 to 1000 mg kg^−1^ CHC was confirmed to improve antioxidant status, and serum lipid metabolites in this study and excess supplementation of CHC is safe in weaning piglets.

## 1. Introduction

Weaning is the most significant event in the life of pigs. Under commercial pig rearing conditions, weaning represents a period of rapid developmental changes that imposes enormous stress on the pig and elicits remarkable changes in gastrointestinal physiology and immunology [1]. During weaning, pigs must cope with the sudden disruption of social interaction with sows and litters and pressure to adapt to new environments that may impair the growth performance and health status of piglets [2]. 

Weaning can induce oxidative stress and result in oxidative injury in pigs [3]. Oxidative stress reflects the imbalance between the systemic phenomenon of reactive oxygen species and the ability of biological systems to detoxify active intermediates, as well as the ability to repair common injuries after weaning [4,5]. Oxidative damage is mainly due to disruption of the oxidant/antioxidant balance, decreased activity of major antioxidant enzymes, free radical-mediated lipid peroxidation, and increased oxidation of proteins and DNA [6]. Bacteria such as *Bacillus coagulans* [7], clinoptilolite [8] and chlorogenic acid [9] have been tried to mitigate oxidative stress in pigs or broilers. Weaning-induced fasting results in mobilization of fat and, to a much lesser extent glycogen, and disturb the balance of lipid metabolism [10]. In a word, optimizing lipid metabolism and protecting the body against oxidative stress play an important role in maintaining good growth performance during these developmental changes at weaning.

Activated charcoal-herb extractum complex (CHC) is a complex of active charcoal and extractum of Chinese herbs (*Pulsatilla chinensis, Portulaca oleracea L., Artemisia argyi Folium* and *Pteris multifida Poir*). Activated charcoal, produced in the presence of an activating reagent, has a high porosity and thus can effectively capture compounds. Chinese herbs, used as natural plant remedies, play an important role in serum lipid metabolism and oxidation capacity. In a previous study, CHC promoted growth performance, alleviated diarrhea, enhanced immune responses, improved intestinal morphology and reduced viable counts of *E. coli* in the cecum [11]. Montmorillonite (MMT) is also widely used as an absorbent for improvement of diarrhea in weaned piglets but did not affect antioxidant status and serum lipid profiles. Excess dietary MMT impairs growth performance and antioxidant capacity in weaned pigs [12]. The effects of CHC in antioxidant status, serum lipid profiles, and safe level of supplementation in weaned pigs is not known.

In the current study, a complex of activated charcoal and herb extractum (CHC) was evaluated to determine its effects on antioxidant status, serum lipid metabolites and safety supplementation in weaned piglets. This CHC complex combines the functions of multiple additives into one product to address several problems encountered in weaned piglets.

## 2. Materials and Methods 

### 2.1. Preparation of Charcoal-Herb Extractum Complex (CHC) and Montmorillonite (MMT)

CHC was obtained from the Fujian Baicaoshaung Biotechnology Co., Ltd (Nanping, China). The manufacture of CHC was reported in a previous paper [11]. In brief, CHC is a combination of activated materials prepared from cedarwood and pine wood and Chinese herbal extractum. The wood was crushed and shifted in particle size, during washing, soaking, water boiling and extraction, and herbs were also sieved to the same size. Finally, the two parts are mixed in appropriate proportions to create CHC. MMT was came from Qingdao Continent Animal Pharmaceutical Co., Ltd. (Shandong, China). CHC had a more developed aperture structure than MMT as shown previously.

### 2.2. Animals, Diets and Experimental Design

Piglets (Duroc × Landrace × Large White), weaned at 28 days of age, were raised at the FengNing Swine Research Unit of China Agricultural University (Academician Workstation in Chengdejiuyun Agricultural and Livestock Co., Ltd) and housed in pens with totally slatted floors (1.2 × 2.0 m). The piglets were grouped completely random design in line with body weights on the day of weaning immediately. Because feeding piglets immediately after weaning is detrimental, we gave less feeding in the first two days of the trial to reduce weaning stress. Each pen was equipped with a stainless-steel feeder and a nipple drinker. All pigs were given ad libitum access to feed and water for 28 days in two phases (days 0–14 and days 15–28). The basal diet was formulated to meet or exceed National Research Council (2012) estimates of the nutrient requirements of weaned pigs (Table 1). All animal procedures and animal care were approved by the Institution Animal Care and Use Committee at China Agricultural University (201605510410554). 

#### 2.2.1. Experiment 1

The purpose of experiment 1 was to evaluate the effects of CHC on antioxidant status and serum lipid metabolites in weaned pigs. Weaned pigs (*n* =216) with an initial body weight of 8.55 ± 1.18 kg were assigned randomly to 6 treatment groups: a corn-soybean meal basal diet supplemented with 500, 1000, 1500 or 2000 mg kg^−1^ of CHC. Negative and positive controls were 0 (CON) and 1000 mg kg^−1^ of MMT (MMT), respectively. Each treatment group had six pens, with six pigs (three barrows and three gilts) per pen. 

We selected 36 pigs near the average group body weight in each pen randomly to collect blood in the morning on d 14 and 28 after 12 h of fasting. Serum samples were separated by centrifugation at 3000 g at 4 °C for 10 min and stored at −20 °C. After 12 h of fasting on the d 28, the pigs which collected the blood were euthanized by electric shock and then bled. Samples of liver and kidney were placed in formalin prior to antioxidant status analysis.

#### 2.2.2. Experiment 2

Experiment 2 was conducted to determine the safety of excess CHC supplementation in weaned pigs. Weaned pigs (*n* = 108) with an initial body weight of 8.58 ± 0.04 kg were assigned randomly to three treatment groups. Each treatment group had six pens (three barrows and three gilts per pen). Pigs were fed a corn-soybean meal-based diet as in Experiment 1 supplemented with 0, 1000 or 10,000 mg kg^−1^ of CHC.

Each piglet was weighed on days 0, 14, and 28 and recorded feed consumption in pens to determine average daily gain (ADG), average daily feed intake (ADFI) and feed to gain ratio (F: G) was calculated. Health data was recorded every day. 18 pigs (1 pig from each pen) were selected randomly to collect blood in the morning on d 14 and 28 after 12 h of fasting. Blood samples were collected from the anterior vena cava and placed in tubes containing ethylene-diaminetetraacetate bi-potassium salt (EDTAK_2_) and no whole blood and serum anticoagulants. Hematological parameters were measured on whole blood within 1 h after sampling. Serum samples were separated by centrifugation at 3000 g at 4 °C for 10 min and stored at −20 °C until analysis. The pigs from which blood had been sampled were euthanized by electrical stunning followed by exsanguination following a 12-h fasting on d 28. Harvest the heart, liver, spleen, lungs and kidneys and weigh them. The relative weight of the heart, liver, spleen, lungs and kidneys is expressed as the ratio to body weight (g kg^−1^). The heart, liver, spleen, lung, kidney and duodenum were sampled and placed in formalin for morphological examination.

### 2.3. Chemical Analysis 

The ingredients and diet were analyzed according to the Association of Official Analytical Chemists’ (AOAC) (2012) procedure [13], including crude protein, total phosphorus and calcium. For analysis of most amino acids, the ingredients and diet were hydrolyzed in 6 M HCl for 24 h at 110 °C. Determination of sulfur amino acid content after formic acid oxidation (AOAC, 2012) was undertaken. Amino acid analysis was carried out using liquid chromatograph (Hitachi L-8800 Amino Acid Analyzer, Tokyo, Japan).

### 2.4. Antioxidant Status

Liver and kidney samples were homogenized in 0.1 mol/L Tris (hydroxymethyl) aminomethane buffer at 4 °C, pH 7.4 to make a 10% (w/v) homogenate, using a Polytron homogenizer for 5 minutes. The ultrasonic homogenizer was centrifuged at 3000 g for 5 minutes at 4 °C for 3 minutes, and the supernatant was collected and stored at −20 °C for enzyme analysis.

In the serum from d 14 and d 28, liver and kidney, the total antioxidant capacity (T-AOC), glutathione peroxidase (GSH-Px), total superoxide dismutase (T-SOD) and malondialdehyde (MDA) concentrations were determined. All assays were performed according to the manufacturer’s instructions using commercial kits (Jiancheng Biochemical Reagent Co., Nanjing, China) [14].

### 2.5. Serum Lipid Metabolites

Serum total cholesterol (TC), triglycerides (TG), high-density lipoprotein (HDL), low-density lipoprotein (LDL) and very low-density lipoprotein (VLDL) concentrations were determined with commercially available kits (Biosino Biotechnology and Science Inc., Beijing, China). The Automatic Biochemical Analyzer was a Hitachi 7160 manufactured by Hitachi High-Tech Corporation (Tokyo, Japan).

### 2.6. Hematological and Serum Biochemical Parameters Analysis

The hematological parameters including white blood cells (WBC), red blood cells (RBC), hemoglobin (HGB), hematocrit (HCT), mean corpuscular volume (MCV), mean corpuscular hemoglobin (MCH), mean corpuscular hemoglobin concentration (MCHC), red cell distribution width (RDW) and platelet count (PLT) were determined using a Sysmex Microcell Counter CL-180 (Tokyo, Japan). Serum biochemical parameters including glucose (GLU), total protein (TP), albumin (ALB), aspartate aminotransferase (AST), alanine aminotransferase (ALT), alkaline phosphatase (ALP), creatinine (CREA) and urea nitrogen (UN), total cholesterol (TC), total triglycerides (TG) and total bilirubin (TBILI) were measured using corresponding commercially available kits (BioSino Bio-technology and Science Incorporated, Beijing, China).

### 2.7. Histopathology Analysis

Tissue samples from heart, liver, spleen, lung, kidney and duodenum were fixed in 10% formalin buffer for 48 h, then dehydrated, cleared and paraffin-embedded. 5 μm thick paraffin sections were stained with hematoxylin and eosin. The change of tissue morphology was observed.

### 2.8. Statistical Analysis 

The data was analyzed as a completely random design using one-way analysis of variance (ANOVA) according to the general liner model (GLM) procedure of SAS 9.2 (SAS Institute Inc., Cary, NC, USA), which included terms for treatment. The pen serves as the experimental unit for growth performance, while the individual pig is the experimental unit for all other indicators. Mean differences were tested using Duncan’s multi-range test. The coefficients of the unequal contrast are generated by the interactive matrix algebra process (IML) of SAS, and then the linear and quadratic responses of the CHC are evaluated using orthogonal polynomials. Significance was declared at *p* < 0.05. An unpaired Student’s *t*-test was used when analyzing the differences between MMT and CHC in the effects of lipid metabolites, MDA and T-SOD. The comparison contrasts were: MMT and CHC 500 mg kg^−1^, MMT and CHC 1000 mg kg^−1^, MMT and CHC 1500 mg kg^−1^, MMT and CHC 2000 mg kg^−1^. * *p* < 0.05, ** *p* < 0.01 were declared as significance. Similarly, the differences between CON and CHC were analyzed with the comparison contrasts of CON and CHC 500 mg kg^−1^, CON and CHC 1000 mg kg^−1^, CON and CHC 1500 mg kg^−1^, CON and CHC 2000 mg kg^−1^. # *p* < 0.05 were declared as significant.

## 3. Results

### 3.1. Experiment 1

#### 3.1.1. Serum Lipid Metabolites 

**Figure 1:**
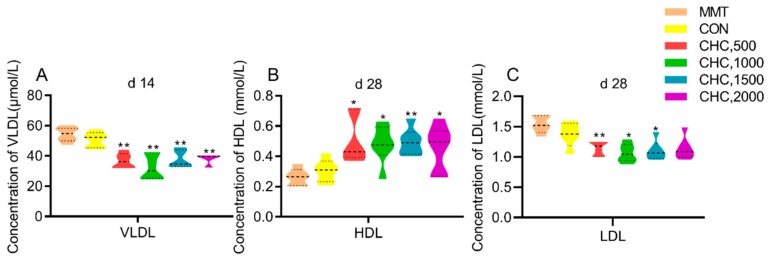
Effects of graded levels of CHC on serum lipid metabolites concentration in weaned piglets (*n* = 6). (**A**) Serum very low-density lipoprotein (VLDL) concentration on d 14. (**B**) Serum high-density lipoprotein (HDL) concentration on d 28. (**C**) Serum low-density lipoprotein (LDL) concentration on d 28. An unpaired Student’s t test was used when analyzing the differences between MMT and CHC in the effects of lipid metabolites. The comparison contrasts were: MMT and CHC 500 mg kg^−1^, MMT and CHC 1000 mg kg^−1^, MMT and CHC 1500 mg kg^−1^, MMT and CHC 2000 mg kg^−1^. * *p* < 0.05, ** *p* < 0.01 were declared as significance. Similarly, the differences between CON and CHC were analyzed with the comparison contrasts of CON and CHC 500 mg kg^−1^, CON and CHC 1000 mg kg^−1^, CON and CHC 1500 mg kg^−1^, CON and CHC 2000 mg kg^−1^.

Figure 1 shows the effects of graded levels of CHC on serum lipid metabolites in weaned piglets. On d 14, concentrations of serum VLDL in CHC supplement groups were lower than negative controls. Compared with the two control groups, pigs fed CHC showed a significantly higher serum HDL concentration. Serum LDL concentration was significantly lower in the pigs fed 500, 1000 and 1500 mg kg^−1^ CHC compared with positive control (MMT) and negative control (CON)s. Other serum lipid traits were not influenced by dietary treatments (Table 2).

#### 3.1.2. Antioxidant Status

**Figure 2:**
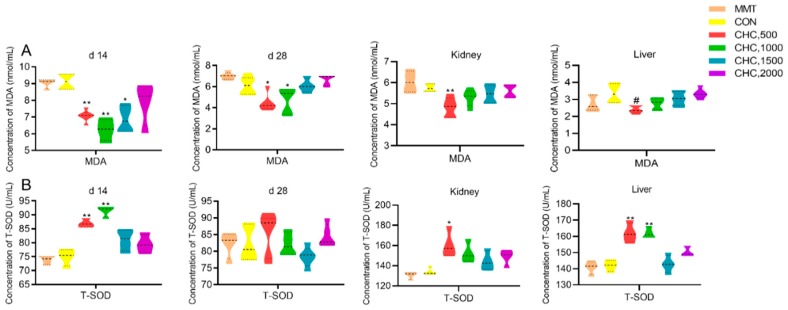
Effects of graded levels of CHC on malondialdehyde (MDA)concentration in weaned piglets (*n* = 6). (**A**) MDA concentration of serum on d 14, d 28, kidney and liver. (**B**) Total superoxide dismutase (T-SOD) concentration of serum on d 14, d 28, kidney and liver. An unpaired Student’s *t*-test was used when analyzing the differences between MMT and CHC in the effects of MDA and T-SOD. The comparison contrasts were: MMT and CHC 500 mg kg^−1^, MMT and CHC 1000 mg kg^−1^, MMT and CHC 1500 mg kg^−1^, MMT and CHC 2000 mg kg^−1^. * *p* < 0.05, ** *p* < 0.01 were declared as significance. Similarly, the differences between CON and CHC were analyzed with the comparison contrasts of CON and CHC 500 mg kg^−1^, CON and CHC 1000 mg kg^−1^, CON and CHC 1500 mg kg^−1^, CON and CHC 2000 mg kg^−1^. # *p* < 0.05 were declared as significant.

We observed pigs fed 500, 1000 and 1500 mg kg^−1^ CHC significantly reduced concentration of serum MDA on d 14 while 500 and 1000 mg kg^−1^ CHC groups had lower concentration of serum MDA on d 28 when compared with MMT and CON (Figure 2). In relation to kidney and liver MDA, 500 mg kg^−1^ CHC group had significantly reduced concentration of MDA in kidney compared with MMT and in liver compared with CON. Similarly, supplementation of CHC at 500 and 1000 mg kg^−1^ significantly increased T-SOD concentration in serum on d 14 and in liver compared with MMT, 500 mg kg^−1^ group significantly increase T-SOD concentration in kidney with MMT. There were no differences in serum, kidney or liver T-AOC and GSH-Px activity among all groups (Table 3).

#### 3.1.3. Serum Biochemical Parameters

There were no significant differences among all groups in the concentration of serum glucose (GLU), total protein (TP), albumin (ALB), aspartate aminotransferase (AST), alanine aminotransferase (ALT), alkaline phosphatase (ALP), creatinine (CREA) and urea nitrogen (UN) on d 14 and d 28 (Table 4).

### 3.2. Experiment 2

#### 3.2.1. Growth Performance and Visceral Index

Compared with the negative control and excess supplementation groups, pigs fed 1000 mg kg^−1^ CHC displayed significantly increased ADG and reduced F: G during the first phase (Table 5). Over the whole experimental period, CHC at 1000 mg kg^−1^ significantly increased body weight and ADG and decreased F: G compared with the negative control (0 mg kg^−1^) and excess supplementation group (10,000 mg kg^−1^) s. When it comes to visceral index, there were no differences in heart, liver, spleen, lung and kidney visceral index among the three experimental groups.

#### 3.2.2. Visceral Organ Histopathology

**Figure 3:**
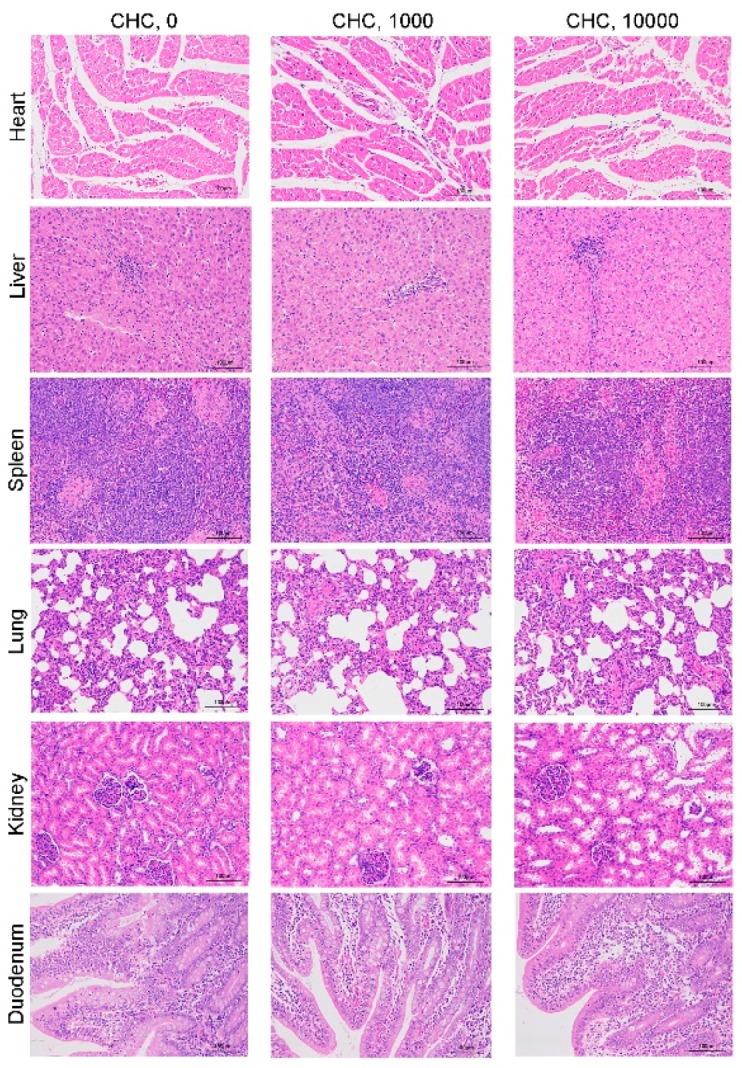
Effects of excess levels of CHC on visceral organ morphology in weaned piglets, scale bars, 100 μm.

Normal histological structures in the heart, liver, spleen, lung, kidney and duodenum were observed in all pigs fed the different diets for 28 days (Figure 3).

#### 3.2.3. Hematological and Serum Biochemical Parameters

On d 28, the high dose of CHC (10000 mg kg^−1^) caused higher ALP level compared to the negative control and the lower dose of CHC (1000 mg kg^−1^). No effect (*p* > 0.05) was observed on hematological parameters (Supplement Table S1) or other serum biochemical parameters (Table 6) among treatments. The results suggest that CHC causes no measurable damage in piglets.

## 4. Discussions

Weaning is the most critical period in the life of young piglets. Nutrition, environment and social factors impact weaned piglets in ways that contribute to accumulated oxidative stress [15]. Early weaning stress is associated with oxidative indicators [16] such as elevated plasma MDA and free radicals and impaired cellular antioxidant defense systems in piglets [17].

In a previous study, we found that CHC can promote growth performance, immunological indices, intestinal morphology and microflora in weaned piglets [11]. In the connect study, we further investigated CHC effects on oxidative stress that generated in weaning piglets and its effect on plasma lipid metabolism. 

Oxidative stress is known as the imbalance between oxidants and detoxification through antioxidant systems. Oxidative stress is traditionally considered harmful to humans because it damages cellular components including lipids, DNA, proteins and carbohydrates, leading to tissue damage [18,19]. Free radicals can produce reactive oxygen species in cells and can cause damage to living cells and tissues [20]. The ability of cells to maintain functional homeostasis depends on the rapid induction of protective antioxidant enzymes. T-SOD in serum may catalyze the dismutation-reaction of free radicals, GSH-Px can remove lipid peroxide, and T-AOC represents the activity of the oxidase system in serum [21]. These antioxidant enzyme activities can effectively protect the structure and functional integrity of cell membranes, and are the main components of important antioxidant systems in the body [22]. Weaning induces oxidative stress in piglets, resulting in oxidative damage in the gastrointestinal track [23]. Thus, it is important to restore and maintain body antioxidant capacity to combine oxidative stress. The antioxidant system owns complete and complex functions comparable to the immune system. Literature has documented how supplementation of antioxidants such as essential oil blends [24], chitosan oligosaccharide [25] and selenomethionine [14] are closely related to improved growth performance in weaning pigs.

In this study, we found that CHC showed its antioxidative effect by decreasing concentration of MDA in serum at 500 to 1000 mg kg^−1^, and kidney and liver MDA at 500 mg kg^−1^. A decreased concentration of MDA in the duodenum and jejunum was observed in pigs fed 500 and 1000 mg kg^−1^ of CHC in previous study [11]. MDA is the end product of lipid peroxidation by free radicals in vivo. A large accumulation of MDA can lead to cross-linking polymerization of proteins, nucleic acids and other living macromolecules. MDA content in vivo can indirectly reflect oxidative stress state of the body [26]. Similarly, we found that CHC increased T-SOD levels in serum. Collectively, these results demonstrated that CHC has beneficial effects on the antioxidant status and protected weaned pigs against oxidative stress. 

Compared with CHC diet and the un-supplemented control, MMT did not influence the antioxidative state of pigs. MMT is a widely used adsorbent for improvement of diarrhea in humans and weaned piglets. It possesses a porous structure with positive charges inside tetrahedral and octahedral layers but negative charges on the surface [27]. MMT higher than 1000 mg kg^−1^ is reported to reduce antioxidant capacity and disrupt liver structure [12]. Lower levels of MMT supplementation are not different from un-supplemented control diets regarding various antioxidant indexes which is in agreement with the current study [12]. 

CHC is a complex of activated charcoal and extractum of Chinese herb medicines, thus its antioxidative activity can be considered separately. The porous structure of activated charcoal allows it to act as an absorbent in the digestive tract. Previous reports document activated charcoal’s chemical efficacy in removing toxins from the gastrointestinal tract of broilers [28,29,30,31] but there are no similar reports with piglets. Toxins are a factor that can generate reactive oxygen species and induce oxidative stress [32]. Absorption of toxins can indirectly reduce the oxidative stress generated by toxins. In this study, we did not examine the effect of activated charcoal independent of the Chinese herb extratum. However, absorption of un favorable components in feedstuffs may have contributed to the improved antioxidative status of CHC-fed pigs.

Four types of Chinese herbs extractum (*Pulsatilla chinensis, Portulaca oleracea L., Artemisia argyi Folium* and *Pteris multifida Poir)* are contained in CHC. They are all widely used as antioxidants. For example, *Portulaca oleracea L.* is linked to strong antioxidant activity and a reduction in blood lipid content [33,34,35] and *Pulsatilla chinensis* can specifically increase superoxide release and increase SOD activity which minimizes superoxide-mediated toxicity [36]. *Artemisia argyi Folium* also plays an important role in mitigating oxidative stress [37].

There are limited reports on the effects of dietary absorbents on lipid metabolites of pigs. Dietary MMT has lipid-adsorbing ability which enhances lipid excretion in feces [17]. Kuusisto [38] reported that patients with hypercholesterolaemia were treated for 4 weeks with activated charcoal and plasma TC and LDL decreased and HDL increased. Our results showed that CHC decreased VLDL and LDL and increased HDL concentration in serum. Increasing concentrations of HDL are strongly associated with decreasing accumulation of atherosclerotic plaques within walls of arteries [39]. Conversely, LDL transports cholesterol from the liver to tissues of the body, and can be oxidized into low-density lipoprotein [40]. Herbs such as *Portulaca oleracea* reduces triglyceridemia, cholesterolemia, and improves reverse cholesterol transport in rats [41], Gatreh-Samani [42] showed that serum TC, TG and LDL decreased and HDL increased in patients who received 50 or 60 g of *Portulaca oleracea* per day. Their researchers indicated that CHC can balance of lipid metabolism capacity and improve lipid composition to good state through the weaning time.

In comparison to other researches evaluating activated charcoal, only 500 to 1000 mg kg^−1^ CHC can improve growth performance and maintain healthy functions superior to the reported. This effective dose was lower than that reported in other studies: 0.25% activated charcoal in African catfish juveniles [43] and 0.3% to 1% bamboo charcoal in fattening pigs [44], chicken [45], Nile tilapia, and ducks [46]. Excessive supplementation of MMT as a mycotoxin adsorbent impairs growth performance, liver function, and antioxidant capacity in starter pigs [12]. A previous study reported that the effective dose of CHC for improvement of growth ranged from 500 to 1000 mg kg^−1^. The purpose of adding a high level (10,000 mg kg^−1^) of CHC, 10 times greater to the highest effective dose, was to evaluate the safety of CHC supplementation in weaning pigs. On d 28, the high dose of CHC (10000 mg kg^−1^) caused a higher ALP level compared to the negative control and the lower dose of CHC (1000 mg kg^−1^). The serum level of ALP in high dose-treated piglets was within the normal range of piglet serum [47]. Excess supplementation of CHC elicited similar growth performance compared with the negative control. Visceral index, visceral organ histopathology and haematological (except ALP) indices were not different from controls when 10,000 mg kg^−1^ CHC were included in the diet. It may be due to the combination of activated charcoal and extractum from Chinese herbs.

## 5. Conclusions

In conclusion, dietary CHC at an inclusion rate of 500 or 1000 mg kg^−1^ is recommended for weaning pigs. This level of supplementation improves the antioxidant status and serum lipid metabolism of weaned pigs. Supplementation of CHC at 10,000 mg kg^−1^ appears to have no detrimental effects on weaned pigs.

## Figures and Tables

**Table 1:** Composition and analyzed nutrient levels of basal diets (%, dry matter basis) ^1^.

Items	Phase I	Phase II
Corn	59.15	60.49
Soybean meal (45% crude protein)	14.31	18.08
Soybean oil	2.80	2.60
Fish meal	2.40	2.20
Soy protein concentrate	10.10	4.80
Whey powder (12% crude protein)	7.32	8.29
Dicalcium phosphate	1.26	1.10
Limestone	0.72	0.60
Salt	0.24	0.24
L-lysine-HCl	0.51	0.50
L-threonine	0.18	0.15
Tryptophan	0.03	0.03
Methionine hydroxy analogue	0.28	0.23
Choline chloride (50%)	0.20	0.20
Vitamine-mineral premix ^2^	0.50	0.50
Total	100.00	100.00
Analyzed nutrient levels		
Digestible energy, Mcal/kg	3.98	3.95
Crude protein	23.50	22.99
Ash	5.55	5.50
Ether extract	6.18	6.34
Crude fiber	3.39	3.37
Lysine	1.72	1.56
Methionine	0.63	0.60
Methionine + Cystine	0.98	0.89
Threonine	1.10	1.01
Calcium	0.85	0.83
Total phosphorus	0.70	0.66

^1^ Corn was replaced with montmorillonite (MMT) or charcoal-herb extractum complex (CHC) in the other treatments. ^2^ Provided per kg of diet: vitamin A, 12,000 IU; vitamin D_3_, 2000 IU; vitamin E, 30 IU; vitamin K_3_, 2.5 mg; thiamine, 2.5 mg; riboflavin, 4 mg; pyridoxine, 3 mg; vitamin B_12_, 20 µg; niacin, 40 mg; pantothenic acid, 12.5 mg; folic acid, 0.7 mg; biotin, 0.07 mg; Fe, 100 mg; Cu, 90 mg; Zn, 80 mg; Mn, 30 mg; I, 0.25 mg; Se, 0.15 mg. Digestible energy values are calculated based on NRC 2012 and other data are analyzed values.

**Table 2:** Effects of graded levels of CHC on serum lipid metabolites concentration in weaned piglets ^1^.

Items	MMT, mg kg^−1^	CHC, mg kg^−1^					SEM	*p* Value		
	1000	0	500	1000	1500	2000		ANOVA	Linear	Quadratic
14 d ^2^										
TC, mmol/L	1.07	1.07	1.30	1.46	1.33	1.58	0.14	0.09	0.25	0.11
TG, mmol/L	0.26	0.28	0.36	0.30	0.39	0.43	0.05	0.20	0.12	0.27
28 d										
TC, mmol/L	1.62	1.56	1.71	1.65	1.82	1.88	0.13	0.52	0.12	0.14
TG, mmol/L	0.26	0.30	0.32	0.27	0.30	0.29	0.04	0.88	0.68	0.66

Note: ^1^ Each mean is the average of 6 observations. Significance was declared at *p* < 0.05. ^2^ MMT montmorillonite, CHC activated charcoal-herb extractum complex, SEM standard error of mean, TC total cholesterol and TG triglyceride.

**Table 3:** Effects of graded levels of CHC on antioxidant status in weaned piglets ^1^.

Items	MMT, mg kg^−1^	CHC, mg kg^−1^					SEM	*p* Value		
	1000	0	500	1000	1500	2000		ANOVA	Linear	Quadratic
Serum 14 d ^2^										
T-AOC, U/mL	13.54	13.39	12.36	11.03	10.18	11.78	1.56	0.62	0.81	0.20
GSH-Px, U/mL	797.52	776.39	760.41	788.71	761.15	787.58	14.51	0.37	0.42	0.60
Serum 28 d										
T-AOC, U/mL	14.68	14.73	14.89	16.04	17.46	12.72	1.48	0.36	0.53	0.44
GSH-Px, U/mL	782.19	798.42	798.04	812.50	816.36	812.78	16.89	0.71	0.99	0.35
Kidney										
T-AOC, U/mg	2.00	2.12	2.39	2.44	2.22	2.20	0.14	0.27	0.19	0.25
GSH-Px, U/mg	449.74	457.20	469.57	466.94	480.30	469.44	25.86	0.97	0.68	0.58
Liver										
T-AOC, U/mg	2.59	2.76	2.68	3.08	2.98	3.25	0.24	0.37	0.91	0.07
GSH-Px, U/mg	606.85	603.72	602.80	667.49	658.37	671.50	40.76	0.63	0.99	0.14

Note: ^1^ Each mean is the average of 6 observations. Significance was declared at *p* < 0.05. ^2^ T-AOC total antioxidant capacity and GSH-Px glutathion peroxidase.

**Table 4:** Effects of graded levels of CHC on serum biochemical indices in weaned piglets ^1^.

Items	MMT, mg kg^−1^	CHC, mg kg^−1^					SEM	*p* Value		
	1000	0	500	1000	1500	2000		ANOVA	Linear	Quadratic
14 d ^2^										
GLU, mmol/L	4.59	4.09	4.26	4.19	4.53	4.62	0.27	0.07	0.18	0.44
TP, g/L	33.72	32.65	38.23	38.67	40.97	46.68	1.04	0.20	0.16	0.12
ALB, g/L	19.88	18.28	21.95	19.72	20.83	24.58	0.16	0.42	0.07	0.48
AST, U/L	37.07	35.92	58.52	37.03	43.03	55.55	8.55	0.26	0.14	0.77
ALT, U/L	23.93	20.67	28.20	26.12	30.03	32.40	2.64	0.06	0.21	0.06
ALP, U/L	257.00	300.43	339.25	325.08	412.87	406.92	46.03	0.16	0.32	0.15
CREA, μmol/L	84.02	83.13	88.43	80.63	89.40	90.25	4.54	0.60	0.11	0.65
UN, mmol/L	2.45	2.93	2.48	2.50	2.91	3.72	0.34	0.11	0.74	0.33
28 d										
GLU, mmol/L	3.68	3.29	3.95	4.83	3.68	4.45	0.36	0.06	0.45	0.15
TP, g/L	39.57	40.90	48.25	45.43	47.30	45.90	2.30	0.07	0.07	0.42
ALB, g/L	27.27	25.92	31.78	30.58	29.97	27.10	1.57	0.09	0.11	0.71
AST, U/L	39.97	43.58	44.33	37.78	33.15	35.38	4.94	0.55	0.96	0.08
ALT, U/L	24.98	27.88	36.58	33.43	33.43	36.48	3.29	0.11	0.06	0.54
ALP, U/L	271.43	284.27	332.00	340.22	324.23	311.12	23.73	0.28	0.36	0.43
CREA, μmol/L	90.27	88.83	105.18	103.85	102.98	103.71	5.65	0.16	0.11	0.28
UN, mmol/L	3.02	2.98	3.38	2.85	3.33	3.43	0.21	0.29	0.07	0.77

Note: ^1^ Each mean is the average of 6 observations. Significance was declared at *p* < 0.05. ^2^ GLU glucose, *TP* total protein, ALB albumin, AST aspartate aminotransferase and ALT alanine aminotransferase, ALP alkaline phosphatase, CREA creatinine and UN urea nitrogen.

**Table 5:** Effects of excess levels of CHC on growth performance and visceral index in weaned piglets ^1^ (n = 6).

Items ^2^	CHC, mg kg^−1^			SEM	*p* Value
	0	1000	10,000		
Body weight					
0 d, kg	8.57	8.58	8.59	0.04	0.97
14 d, kg	12.81 ^b^	13.45 ^a^	12.31 ^b^	0.17	<0.01
28 d, kg	20.57 ^b^	21.54 ^a^	20.20 ^b^	0.28	0.02
0–14 d					
ADG, kg	0.30 ^b^	0.35 ^a^	0.27 ^b^	0.01	<0.01
ADFI, kg	0.53 ^a^	0.55 ^a^	0.46 ^b^	0.02	0.02
F: G	1.74 ^a^	1.57 ^b^	1.75 ^a^	0.03	<0.01
15–28 d					
ADG, kg	0.55	0.58	0.57	0.02	0.63
ADFI, kg	0.86	0.86	0.85	0.03	0.96
F: G	1.57	1.48	1.50	0.06	0.54
0–28 d					
ADG, kg	0.43 ^ab^	0.46 ^a^	0.41 ^b^	0.01	<0.05
ADFI, kg	0.70	0.70	0.66	0.02	0.36
F: G	1.63 ^a^	1.51 ^b^	1.59 ^a^	0.03	0.04
Visceral index, g kg^−1^					
Heart	5.62	5.57	5.60	0.21	0.99
Liver	29.12	27.67	28.70	1.31	0.73
Spleen	2.45	2.28	2.60	0.17	0.47
Lung	11.14	11.04	11.48	0.54	0.84
Kidney	5.27	5.29	5.70	0.26	0.43

Note: ^1, a, b^ Means within the same row without common superscripts differ significantly (*p* < 0.05). ^2^ BW body weight, ADG average daily gain, ADFI average daily feed intake, F: G feed to gain ratio.

**Table 6:** Effects of excess levels of CHC on serum biochemical indexes in weaned piglets ^1^.

Items ^2^	CHC, mg kg^−1^			SEM	*p* Value
	0	1000	10,000		
14 d					
GLU, mmol/L	4.40	4.07	4.04	0.19	0.46
TP, g/L	34.48	38.08	44.83	3.54	0.18
ALB, g/L	28.35	29.53	33.23	2.45	0.38
AST, U/L	36.92	38.28	43.75	8.34	0.83
ALT, U/L	22.12	25.53	31.00	2.63	0.10
ALP, U/L	261.67	346.83	378.48	36.14	0.11
CREA, μmol/L	84.87	80.62	89.53	5.18	0.50
UN, mmol/L	2.96	2.67	3.45	0.33	0.30
TC, mmol/L	1.07	1.39	1.26	0.09	0.07
TG, mmol/L	0.31	0.29	0.40	0.06	0.42
TBILI, μmol/L	0.85	0.80	0.80	0.13	0.95
28 d					
GLU, mmol/L	3.93	4.28	4.83	0.30	0.14
TP, g/L	60.28	65.50	61.35	1.80	0.15
ALB, g/L	23.88	28.73	27.02	1.24	0.06
AST, U/L	33.35	35.73	34.68	3.97	0.91
ALT, U/L	33.02	35.27	32.27	2.41	0.67
ALP, U/L	221.27 ^b^	312.33 ^a^	325.52 ^a^	25.29	0.03
CREA, μmol/L	89.72	105.22	98.75	5.17	0.15
UN, mmol/L	3.47	3.35	3.23	0.23	0.76
TC, mmol/L	1.29	1.37	1.40	0.10	0.76
TG, mmol/L	0.30	0.28	0.38	0.05	0.32
TBILI, μmol/L	1.20	1.07	0.95	0.13	0.43

Note: ^1 a, b^ Means within the same row without common superscripts differ significantly (*p* < 0.05). ^2^ Each mean is the average of 6 observations. Mean differences were tested using Duncan’s multi-range test. The coefficients of the unequal contrast are generated by the interactive matrix algebra process (IML) of SAS. Significance was declared at *p* < 0.05. ^3^ GLU glucose, TP total protein, ALB albumin, AST aspartate aminotransferase, ALT alanine aminotransferase, ALP alkaline phosphatase, CREA creatinine, UN urea nitrogen, TC total cholesterol, TG total triglycerides and TBILI total bilirubin.

## Supplementary Materials

The following are available online at https://www.mdpi.com/2076-2615/9/12/1151/s1.
