# Supplementary material for: Effects of Activated Charcoal-Herb Extractum Complex on Antioxidant Status, Lipid Metabolites and Safety of Excess Supplementation in Weaned Piglets
Source: Animals (Basel). 2019 Dec 15;9(12):1151. doi: 10.3390/ani9121151 (PMC6940724; doi:10.3390/ani9121151)
Supplement: Supplementary file 1 [file animals-09-01151-s001.pdf]

Table S1. Effects of excess levels of CHC on hematological indexes in weaned piglets [1].

**Item** [2]	**CHC, mg kg^{-1}**			**SEM**	***p* Value**
	**0**	**1000**	**10,000**		
14 d					
WBC, 10^9/L	20.37	22.15	22.87	1.84	0.63
RBC, 10^{12}/L	6.10	5.78	6.20	0.13	0.10
HGB, g/L	105.00	102.50	109.17	2.59	0.23
HCT, %	33.15	32.07	32.05	1.45	0.83
MCV, fL	55.02	56.13	55.38	0.96	0.71
MCH, pg	17.40	17.58	17.50	0.32	0.92
MCHC, g/L	309.33	313.17	313.33	2.54	0.48
RDW, %	21.33	20.10	21.23	1.15	0.71
PLT, 10^9/L	345.33	367.67	342.83	29.40	0.81
MPV, fL	10.23	10.15	10.93	0.55	0.56
PDW, fL	17.10	17.42	18.17	0.42	0.23
28 d					
WBC, 10^9/L	20.58	21.12	20.52	1.94	0.97
RBC, 10^{12}/L	5.98	5.68	5.81	0.26	0.73
HGB, g/L	105.33	99.17	102.17	4.96	0.69
HCT, %	33.28	31.13	32.33	1.62	0.65
MCV, fL	57.78	57.32	56.57	1.48	0.85
MCH, pg	17.93	17.42	17.43	0.50	0.72
MCHC, g/L	308.33	304.67	303.83	3.44	0.63
RDW, %	20.97	20.32	21.62	1.04	0.69
PLT, 10^9/L	251.83	286.83	280.00	21.41	0.50
MPV, fL	9.87	9.73	9.22	0.36	0.44
PDW, fL	16.52	17.03	17.17	0.37	0.46

Note: [1] Each mean is the average of 6 observations. [2] WBC white blood cells, RBC red blood cells, HGB hemoglobin, HCT hematocrit, MCV mean corpuscular volume, MCH mean corpuscular hemoglobin, MCHC mean corpuscular hemoglobin concentration, RDW red cell distribution width, PLT platelet count, MPV mean platelet volume and PDW platelet distribution width.
